# Guidelines and variations in patterns of GnRH analogue use in castration-resistant prostate cancer across six countries

**DOI:** 10.1016/j.esmorw.2025.100122

**Published:** 2025-03-04

**Authors:** G. George, D. Enting, H. Garmo, P. Stattin, I.F. Lissbrant, M. Monroy-Iglesias, L.-M. Scailteux, F. Balusson, C. Van Praet, N. Lumen, G. Marvaso, G. Corrao, B.A. Jereczek-Fossa, L. Chehade, A. Shamseddine, M. Charafeddine, M. Van Hemelrijck

**Affiliations:** 1 Transforming cancer OUtcomes through Research (TOUR), School of Cancer and Pharmaceutical Studies, King’s College London, London, UK; 2 Department of Oncology, Guy’s and St Thomas’ NHS Foundation Trust, London, UK; 3 Department of Surgical Sciences, Uppsala University Hospital, Uppsala, Sweden; 4 Department of Oncology, Institute of Clinical Sciences, The Sahlgrenska Academy, Gothenburg, Sweden; 5 University Rennes, CHU Rennes, Inserm, EHESP, Irset (Institut de recherche en santé, environnement et travail)—UMR_S 1085, Rennes, France; 6 Department of Urology, Ghent University Hospital, Ghent, Belgium; 7 Department of Oncology and Haematology-Oncology, University of Milan, Milan, Italy; 8 Department of Radiotherapy, European Institute of Oncology (IEO), IRCCS, Milan, Italy; 9 Department of Internal Medicine, Division of Haematology-Oncology, American University of Beirut Medical Center, Beirut, Lebanon

**Keywords:** castration-resistant prostate cancer, GnRH analogues, real-world data, cohort, prostate cancer treatment

## Abstract

**Background:**

We investigated patterns of gonadotropin-releasing hormone (GnRH) analogue use in castration-resistant prostate cancer (CRPC) using real-world data from six countries.

**Patients and methods:**

Data were obtained from Guy’s and St Thomas’ NHS Foundation Trust (GSTT, UK), Prostate Cancer data Base Sweden (PCBaSe RAPID 2019, Sweden), Système National Des Données De Santé (SNDS, France), European Institute of Oncology (IEO, Italy), Ghent University Hospital (GUH, Belgium), and American University of Beirut Medical Center (AUB, Lebanon). Men diagnosed with CRPC between 2017 and 2019 were included in the study, with data extended to 2020 where available. Cox proportional hazards regression models were used to assess the effect of discontinuing GnRH analogues on overall mortality, adjusting for: age, Charlson Comorbidity Index (CCI), initial prostate cancer (PCa) treatment, and PCa risk group.

**Results:**

Out of 24 141 men with CRPC across the six countries, 1367 patients discontinued GnRH analogues. The discontinuation rates varied by country, with notable proportions from Sweden (338 men, 13%), France (1024 men, 5%), and Lebanon (5 men, 13%). Following adjustment for age, CCI, initial treatment for PCa, and PCa risk group, a higher risk of mortality in men who discontinued GnRH analogues was observed in Sweden (hazard ratio 2.30, 95% confidence interval 2.03-2.62).

**Conclusions:**

Our study showed that although guidelines recommend the continued use of GnRH analogues in men with CRPC, a small proportion of men discontinue, the reasons for which are not fully understood. Further research is needed to explore potential contributing factors, such as differences in clinical practices, patient characteristics, or health care system across the countries studied.

## Highlights

• Differences in GnRH use may inform policy changes to improve patient outcomes.
• The study aimed to evaluate treatment patterns in patients who continue or discontinue as prescribed.
• Guidelines advise CRPC men to continue GnRH, a minority stop due to side-effects.
• Variability in data sources made it difficult to define discontinuation accurately.
• Variabilities were temporary/complete discontinuation or switch between GnRH types.

## Introduction

Castration-resistant prostate cancer (CRPC) is defined by progression of prostate cancer (PCa) despite androgen deprivation therapy (ADT). This indicates that the cancer has developed mechanisms to grow even in a low-androgen environment, making it resistant to standard hormonal treatments. CRPC is characterised by prostate-specific antigen (PSA) levels rising, new metastases, or worsening symptoms despite castration-level testosterone. Although it is a more advanced and difficult stage of PCa to treat, the disease may still respond to additional therapies that target androgen receptors or androgen production. The currently approved agents for the treatment of CRPC maintain ADT, gonadotropin-releasing hormone (GnRH) agonists, with other therapies such as chemotherapy or androgen receptor-targeted agents, which will depend on the disease’s metastatic status.^1^

The evidence for continuing ADT in CRPC remains debatable among clinicians. Findings from O’Donnell et al. and randomised prospective data support ADT maintenance with abiraterone therapy in CRPC because abiraterone monotherapy does not sustain testosterone in non-castrated men.^2^ Moreover, phase III trial data on enzalutamide in CRPC also suggests ADT continuation as the backbone because of the potential gynaecomastia as a side-effect of enzalutamide monotherapy.^3^ Increasing evidence indicates that even when ADT suppresses serum testosterone levels, androgen activity persists inside the prostate and PCa microenvironment, which suggests that including ADT in combination treatments may still be required in CRPC.^4^ However, treatment with abiraterone acetate plus prednisone (AA + P) without GnRH, compared with AA + P and GnRH, has shown to reduce serum testosterone levels in the COU-AA 302 phase III clinical trial.^5^ In castrated patients, AA + P treatment without GnRH resulted in higher PSA response rates, longer duration to PSA progression, and radiographic progression-free survival, which was also supported by the results of an exploratory German phase II trial, SPARE, that investigated AA + P versus AA + P and GnRH.^6^

Understanding differences in patterns of GnRH analogue use across countries despite uniform guidelines can provide valuable insights into health care practices, identify potential gaps in treatment patterns, and help lead policy changes to promote the best possible patient outcomes. By leveraging real-world data from health care databases in the United Kingdom (UK), Sweden, France, Italy, Belgium, and Lebanon, we investigated patterns of GnRH analogue use in men with CRPC using real-world data from six different countries. The aim was to identify and evaluate the treatment patterns of patients who either continue or discontinue for the prescribed duration.

## Patients and methods

All men diagnosed with CRPC between 2017 and 2019 (follow-up until 2020) were included with no further exclusion criteria. Exposure was defined from filled prescription date for any GnRH analogue. The outcome was defined as GnRH status (continued or discontinued) at the censor date, which was determined as either the unplanned GnRH stop date or date of death or the end of the study, whichever occurred first. Hospital data were obtained from Guy’s and St Thomas’ NHS Foundation Trust (GSTT) in the UK, European Institute of Oncology (IEO) in Italy, Ghent University Hospital (GUH) in Belgium, and American University of Beirut Medical Center (AUB) in Lebanon. Definitions from a study protocol were applied to extract data for each hospital dataset at the respective sites. In Sweden, national-level data were extracted from Prostate Cancer data Base Sweden (PCBaSe) RAPID 2019. PCBaSe offers a comprehensive, register-based resource with linkages to the National Prostate Cancer Register providing detailed information on tumour characteristics, treatments, comorbidities, and outcomes.^7^ A claims insurance database, Système National Des Données De Santé (SNDS), was used in France which covers all health care expenses reimbursed by the French health insurance system, with detailed information on demographics, medical diagnoses, treatments, hospitalisations, and mortality data.^8^ The research ethics board in Uppsala approved the study for PCBaSe. The French Data Protection Authority (CNIL) approved the use of the SNDS (DR-2021-147). Local ethics committees approved the use of IEO in Italy (IEO 1746), GUH in Belgium (UZG 2015/0260), and AUB in Lebanon.

A detailed study protocol was used to ensure uniformity across the data sources used in this study. To minimise disparities arising from differences in data collection methods between the datasets, the protocol outlined detailed definitions for study variables (Table 1 includes detailed classifications) used for data extraction and analysis. A study coordinator oversaw data collection across all sites, ensuring adherence to the standardised study protocol, and analysed all data except for France, where data sharing regulations applied.

**Table 1:** Characteristics of men with castration-resistant prostate cancer in the UK, Sweden, France, Italy, Belgium, and Lebanon

Patient characteristics	UK	Sweden	France	Italy	Belgium	Lebanon
n	450	2700	20 843	50	58	40
GnRH status, n (%)						
Yes (GnRH continued)	450 (100)	2362 (87.5)	19 819 (95.1)	50 (100)	58 (100)	35 (87.5)
No (GnRH discontinued)	0	338 (12.5)	1024 (4.9)	0	0	5 (12.5)
Mean age, years (SD)	72.7 (9.3)	75.3 (8.0)	76.9 (9.2)	70.0 (8.9)	72.4 (10.6)	71.9 (9.9)
Mean follow-up time, years (SD)	7.2 (5.1)	1.9 (1.2)	1.39 (0.96)	2.5 (1.2)	1.5 (1.1)	2.8 (1.4)
Risk group at diagnosis, n (%)						
Low risk (cT1-2a, PSA 10 ng/ml, and Gleason 7)		162 (6.0)	NA	0	NA	1 (2.5)
Intermediate risk (cT2b or PSA 10-20 ng/ml or Gleason 7)	6 (1.3)	429 (15.9)	NA	6 (12.0)	NA	9 (22.5)
High risk (cT2c or PSA 20 ng/ml or Gleason 7)	58 (12.9)	395 (14.6)	NA	19 (38.0)	NA	7 (17.5)
High risk (not localised)		573 (21.2)	NA		NA	
Locally advanced/metastatic (patients with nodal or metastatic disease)	337 (74.9)	1124 (41.6)	NA	23 (46.0)	NA	22 (55.0)
Missing	49 (10.9)	17 (0.6)	NA	2 (4.0)	NA	1 (2.5)
Initial PCa treatment, n (%)						
None or deferred treatment	14 (3.1)	276 (10.2)	0	0	1 (1.7)	0
Radical prostatectomy		402 (14.9)		7 (14.0)		9 (22.5)
Hormonal therapy	31 (6.9)	1550 (57.4)	19 058 (91.4)	18 (36.0)	19 (32.8)	23 (57.5)
Radiotherapy	274 (60.9)	394 (14.6)	744 (3.6)	2 (4.0)	1 (1.7)	1 (2.5)
Radical prostatectomy and hormonal therapy or radical prostatectomy and radiotherapy	21 (4.7)	0	68 (0.3)	19 (38.0)	9 (15.5)	2 (5.0)
Radiotherapy and hormonal therapy	12 (2.7)	0	346 (1.7)	4 (8.0)	3 (5.2)	5 (12.5)
Chemotherapy and hormonal therapy	41 (9.1)	0	603 (2.9)	0	16 (27.6)	0
Chemotherapy and radiotherapy and hormonal therapy	57 (12.7)	0	24 (0.1)	0		0
Missing	0	78 (2.9)	0	0	9 (15.5)	0
Hypertension, n (%)						
Yes	199 (44.2)	NA	13 560 (65.1)	17 (34.0)	37 (63.8)	20 (50)
No	251 (55.8)	NA	7283 (34.9)	33 (66.0)	17 (29.3)	20 (50)
Missing	0	NA	0	0	4 (6.9)	0
Diabetes, n (%)						
Yes	71 (15.8)	NA	4532 (21.7)	4 (8.0)	13 (22.4)	7 (17.5)
No	379 (84.2)	NA	16 311 (78.3)	46 (92.0)	41 (70.7)	33 (82.5)
Missing	0	NA	0	0	4 (6.9)	0
Dyslipidaemia, n (%)						
Yes	79 (17.6)	NA	8563 (41.1)	1 (2.0)	17 (29.3)	8 (20.0)
No	371 (82.4)	NA	12 280 (58.9)	49 (98.0)	37 (63.8)	32 (80.0)
Missing	0	NA	0	0	4 (6.9)	0
Cardiovascular diseases, n (%)						
Yes	54 (12.0)	NA	3453 (16.6)	4 (8.0)	19 (32.8)	9 (22.5)
No	396 (88.0)	NA	17 390 (83.4)	46 (92.0)	35 (60.3)	31 (77.5)
Missing	0	NA	0	0	4 (6.9)	0
CCI group, n (%)						
0	0	1909 (70.7)	0	0	0	0
1-3	289 (64.2)	739 (27.4)	14 221 (68.2)	1 (2.0)	0	0
≥4	154 (34.2)	52 (1.9)	6622 (31.8)	49 (98.0)	58 (100)	40 (100)
Missing	7 (1.6)	0	0	0	0	0

ATC, anatomical therapeutic chemical; CCI, Charlson Comorbidity Index; GnRH, gonadotropin-releasing hormone; ICD, International Classification of Diseases; NA, not available; PCa, prostate cancer; PSA, prostate-specific antigen; SD, standard deviation.

Hypertension (ICD-10: I10 + ATC for drugs); diabetes (ICD-10: E10-E14 + ATC for drugs); dyslipidaemia (ICD-10: E78 + ATC for drugs); CVD (ICD-10: I20-I99, G45).

Patient characteristics of men with CRPC in the UK, Sweden, France, Italy, Belgium, and Lebanon were expressed as proportions (Table 1). Cox proportional hazards regression was used to investigate the effect of GnRH analogue discontinuation on overall mortality. Models were adjusted for: (i) age and Charlson Comorbidity Index (CCI); (ii) age, CCI, initial treatment for PCa; and (iii) age, CCI, initial treatment for PCa, and risk group at PCa diagnosis. This was expressed as hazard ratios (HRs) and 95% confidence intervals (CIs).

All analyses were conducted using Software for Statistics and Data Science (STATA) version 15 (StataCorp LLC, College Station, TX).

## Results

Men with CRPC continued GnRH analogues at the end of study period in the UK, Italy, Belgium, and Lebanon. The overall median follow-up time for men with CRPC in the six countries was 2.2 years (interquartile range 1.3 years). Since no men discontinued in the UK, Italy, and Belgium and only five men discontinued in Lebanon, no analysis was conducted. However, analyses were conducted for men with CRPC in Sweden and France because 13% of men in Sweden and 5% of men in France with CRPC discontinued GnRH at the end of the study period.

In Sweden (Table 2), crude HRs from Cox proportional hazards models showed a higher risk of mortality in those who discontinued GnRH in PCBaSe compared with men with CRPC who continued GnRH analogues (HR 2.19, 95% CI 1.94-2.48). This pattern remained consistent upon adjustment to age and CCI group (HR 2.35, 95% CI 2.08-2.67); age, initial treatment for PCa, and CCI (HR 2.33, 95% CI 2.05-2.65); and age, initial treatment, CCI, and risk group at diagnosis (HR 2.30, 95% CI 2.03-2.62).

**Table 2:** HRs and 95% CIs for men with castration-resistant prostate cancer who discontinue GnRH analogues in Sweden and France

	Sweden				France		
	Crude HR (95% CI)	Adjusted HR (95% CI)^a^	Adjusted HR (95% CI)^b^	Adjusted HR (95% CI)^c^	Crude OR (95% CI)	Adjusted HR (95% CI)^a^	Adjusted HR (95% CI)^b^
GnRH continuers	1.00 (Ref)	1.00 (Ref)	1.00 (Ref)	1.00 (Ref)	1.00 (Ref)	1.00 (Ref)	1.00 (Ref)
GnRH discontinuers	2.19 (1.94-2.48)	2.35 (2.08-2.67)	2.33 (2.05-2.65)	2.30 (2.03-2.62)	1.29 (1.12-1.49)	1.06 (0.92-1.22)	1.06 (0.92-1.22)
Mean age	—	1.03 (1.02-1.04)	1.02 (1.02-1.03)	1.03 (1.02-1.03)	—	1.07 (1.06-1.07)	1.07 (1.06-1.07)
CCI group							
0	—	1.00 (Ref)	1.00 (Ref)	1.00 (Ref)	—	1.00 (Ref)	1.00 (Ref)
1-3	—	1.11 (1.00-1.24)	1.13 (1.01-1.26)	1.14 (1.02-1.28)	—	0.92 (0.76-1.12)	0.92 (0.76-1.12)
≥4	—	2.34 (1.70-3.22)	2.32 (1.68-3.20)	2.27 (1.64-3.15)	—	2.57 (2.11-3.13)	2.56 (2.12-3.14)
Initial treatment for PCa							
Hormonal therapy	—	—	1.00 (Ref)	1.00 (Ref)	—	—	1.00 (Ref)
None or deferred treatment	—	—	0.98 (0.83-1.16)	0.99 (0.80-1.23)	—	—	—
Radical prostatectomy	—	—	0.63 (0.54-0.75)	0.66 (0.53-0.81)	—	—	0.70 (0.29-1.68)
Radiotherapy	—	—	0.68 (0.58-0.79)	0.72 (0.61-0.86)	—	—	0.79 (0.65-0.95)
Risk group at PCa diagnosis							
Low risk (cT1-2a, PSA 10 ng/ml, and Gleason 7)	—	—	—	1.00 (Ref)	—	—	—
Intermediate risk (cT2b or PSA 10-20 ng/ml or Gleason 7)	—	—	—	1.00 (0.78-1.29)	—	—	—
High risk (cT2c or PSA 20 ng/ml or Gleason 7)	—	—	—	1.00 (0.76-1.31)	—	—	—
High risk (not localised)	—	—	—	0.91 (0.69-1.19)	—	—	—
Locally advanced/metastatic (patients with nodal or metastatic disease)	—	—	—	1.07 (0.81-1.41)	—	—	—

CCI, Charlson Comorbidity Index; CI, confidence interval; GnRH, gonadotropin-releasing hormone; HR, hazard ratio; PCa, prostate cancer; PSA, prostate-specific antigen.

a Adjusted: adjusted for age and CCI group.

b Adjusted: adjusted for age, initial treatment for PCa, and CCI.

c Adjusted: adjusted for age, initial treatment, CCI, and risk group at diagnosis.

Although crude analysis in France showed a higher risk of mortality in men who discontinued GnRH compared with men with CRPC who continued GnRH analogues (HR 1.29, 95% CI 1.12-1.49), this was no longer statistically significant following adjustments (Table 2).

## Discussion

Our study showed that even though guidelines recommend the continued use of GnRH analogues in men with CRPC, a small proportion of men (5%-13% in Sweden and France) discontinue. The findings highlight the need for individualised treatment strategies and enhanced monitoring of CRPC patients who discontinue GnRH analogues. While continuous ADT is generally recommended, some men with CRPC may benefit from tailored approaches, especially considering the potential side-effects of long-term GnRH use, such as cardiovascular complications.

The increased side-effects of additional cancer therapies or disease progression at the time of CRPC diagnosis may be one factor contributing to the discontinuation of GnRH analogues observed in men with CRPC. The addition of other cancer therapies along with GnRH analogues may lead to cumulative toxicities.^1^^,^^9^^,^^10^ Therefore, men with CRPC may find it easier to discontinue GnRH analogues temporarily for a period until they start to cope with combined impact of side-effects from new treatment regimens and disease progression. Moreover, the pattern of GnRH discontinuation and increased mortality observed in Sweden may be attributed to the possibility that men with CRPC in the palliative setting may be more likely to discontinue GnRH analogues to alleviate the side-effects.

The risks associated with GnRH discontinuation in the CRPC setting remain an area to be explored. A single-arm, phase II clinical trial investigated the effects of using abiraterone without continued use of GnRH analogues in patients with metastatic PCa.^11^ This study showed that men with metastatic PCa given AA + P after GnRH analogue discontinuation remained castrate 30 months after GnRH discontinuation. Although not in the CRPC setting, Makrakis et al. provide some evidence to support the safety and efficacy of GnRH discontinuation in metastatic PCa, serving as a starting point for further exploration of this regimen in the CRPC setting.

While this study provides valuable insights, there were some limitations that need to be considered when interpreting the results. Firstly, the use of various types of data sources including national registries, claims databases, and hospital databases introduced variability that made it challenging to accurately distinguish between temporary discontinuation, complete discontinuation, and the switching between different GnRH analogues. Secondly, the lack of adjustment for GnRH duration may have influenced clinical outcomes, as men on prolonged treatment are less likely to recover serum testosterone levels after discontinuation. Thirdly, the larger French cohort showed no negative effects from GnRH discontinuation in the Cox analysis. The observed differences in results between Sweden and France may thus be partially explained by variations in baseline characteristics, such as higher CCI and the use of hormonal therapy as the initial treatment for PCa, data coding systems, as well as differences in treatment duration. Finally, while overall survival was the primary endpoint in our analysis, PCa-specific survival may offer additional insights into the effects of GnRH discontinuation, particularly in men with extended exposure to treatment. This work highlights that when using real-world data, homogeneous data collection from heterogeneous databases is necessary to address implementation-focused clinical research issues like adherence to clinical recommendations.

Therefore, it is recommended that the backbone ADT is maintained when initiating chemotherapy or other therapies in the CRPC setting. Even though additional cancer therapies or increased adverse effects from cancer progression may partially account for GnRH discontinuation, further qualitative research is needed to fully understand the reasons for GnRH discontinuation in the CRPC setting.
